# Clinically-diagnosed vitamin deficiencies and disorders in the entire United States military population, 1997–2015

**DOI:** 10.1186/s12937-021-00708-2

**Published:** 2021-06-15

**Authors:** Joseph J. Knapik, Emily K. Farina, Victor L. Fulgoni, Harris R. Lieberman

**Affiliations:** 1 Military Nutrition Division, US Army Research Institute of Environmental Medicine, 10 General Greene Ave, Natick, MA 01760 USA; 2 Nutrition Impact, LLC, 9725 D Drive North, Battle Creek, MI 49014 USA

**Keywords:** Hypervitaminosis, Hypovitaminosis, Sex, Age, Race, Military service

## Abstract

**Background:**

This study examined incidence rates, temporal trends, and demographic factors associated with vitamin deficiencies/disorders in all United States military personnel from 1997 to 2015 (mean *N* = 1,382,266/year).

**Methods:**

Employing an ecological study design, the Defense Medical Epidemiological Database and specific International Classification of Diseases codes were used to determine incidence rates for clinically-diagnosed vitamin deficiencies/disorders. Associations with demographic factors were examined.

**Results:**

The overall incidence rate of vitamin deficiencies/disorders was 92.7 cases/100,000 person-years (p-yr). Highest rates were for vitamin D (53.7 cases/100,000 p-yr), other B-complex vitamins (20.2 cases, 100,000 p-yr), vitamin B_12_ anemia (7.6 cases/100,000 p-yr), deficiencies of “other vitamins” (5.9 cases/100,000 p-yr), and vitamin A (2.5 cases/100,000 p-yr). Thiamin, riboflavin, niacin, pyridoxine, folate, vitamin C, and vitamin K deficiencies and hypervitaminoses A and D had < 1 case/100,000 p-yr. Rates for vitamin D, other B-complex, “other vitamin”, and thiamin deficiencies increased over time, while vitamin A and C deficiencies decreased. Women had higher incidence rates for all examined deficiencies/ disorders except niacin and vitamin C. Incidence rates rose with age in 8 of 15 deficiency/disorder categories and blacks had higher incidence rates in 9 of 15 deficiency/disorder categories.

**Conclusions:**

The overall rate of clinically-diagnosed vitamin deficiencies and disorders was low but higher in women and minority subgroups. As for most illnesses, the diagnosed incidence of such disorders may be an underestimate of the actual incidence. These findings can guide clinical decision making with regard to testing for nutritional deficiencies and delivering public health information to at risk populations.

**Clinical trial registration:**

(No. ISRCTN58987177). Registration date 9 October 2019.

## Background

Information from medical records on the incidence of clinically-diagnosed dietary deficiencies and hypervitaminoses in a representative sample of the United States (US) population is not available. Nutritional deficiencies are of special interest to the armed forces since they have occurred in various military organizations throughout history, compromising the health and operational effectiveness of service members (SMs). For example, in the 18th and 19th centuries, many sailors in the British Royal Navy were disabled by scurvy until it was discovered the disease was caused by a diet lacking fresh fruits and vegetables, thereby resulting in ships being provisioned with these foods [1]. Many years later, vitamin C deficiency was found to be the underlying cause of the disease [2]. During the US Civil War, a lack of appreciation that vitamin A deficiency resulted in night blindness caused many physicians to ascribe this medical condition to malingering [3]. With improved knowledge of the importance of nutrition, enrichment of certain foods, and dramatic increases in living standards, diseases resulting from nutritional deficiencies or excess and other nutrition-related maladies have been greatly reduced in civilian and military populations in developed countries, but can still be present, and affect health and performance [4–7].

The most common vitamin deficiencies in the US are those associated with vitamin D and vitamin B_12_. Vitamin D is essential for maintaining bone health and performs other important metabolic functions [7–10]. The incidences of vitamin D deficiency were 24 and 54% in healthy adolescents and community dwelling elderly homebound individuals, respectively [11, 12]. Lower levels of 25(OH) D have been found to be associated with increased risk of stress fractures in a number of military investigations [13] and Special Operations soldiers have reduced dietary quality, including reduced vitamin D intake when deployed [14]. Vitamin B_12_ is essential for the synthesis of DNA, blood formation, and brain and nerve health [15, 16]; deficiency can lead to hematological, neuropsychiatric, and cardiovascular disorders [17]. Based on data from the 1999 to 2002 National Health and Nutrition Examination Survey (NHANES), 3–6% of Americans were B_12_ deficient while 14–20% had marginal B_12_ depletion [18]. B_12_ deficiency increases with age [18] and can be especially high among adult vegetarian and vegans where deficiency prevalences ranging from 32-90%, have been reported [19]. Other data [20] indicate that prescriptions filled by military SMs for vitamin C and D have risen over a recent 10-year period suggesting military medical care providers believe that supplementation with certain vitamins is essential to maintain the optimal health of SMs.

We are not aware of any studies that have used medical records to assess the incidence of vitamin deficiencies in any civilian or military population, although the NHANES does provide detailed information on various biomarkers of nutritional status in a representative sample of the US population [21]. The US Department of Defense maintains an epidemiological database that contains all medical encounters of SMs, the International Classification of Diseases Ninth Revision (ICD-9) diagnosis codes associated with each encounter, and basic demographic data. By using these data, the incidence of specific diagnosed vitamin deficiencies can be determined for the total diverse population of SMs, consisting of over 1 million individuals. Military populations may be more susceptible to vitamin deficiencies and disorders due to their frequent deployment to harsh environments, high levels of physical activity, and limited availability of fresh food in some situations. Therefore, this study examined incidence rates, temporal trends, and demographic factors associated with vitamin deficiencies/disorders in all US military personnel. We hypothesized that these factors would differ depending on the vitamins examined.

## Methods

The US Army Research Institute of Environmental Medicine Human Research Protection Office approved this ecological (population-based) study of de-identified data from the Defense Medical Epidemiological Database (DMED). The DMED is an online database that contains inpatient and outpatient medical encounters of all uniformed military personnel (Army, Navy, Marine Corps, and Air Force). The data in the DMED include ICD-9 codes that medical care providers enter into the system after they have assessed the patient during the encounter and determined a diagnosis. By using the DMED, the number of any single medical diagnosis per year in the military population can be determined based on ICD-9 codes. The incidence rates of these medical diagnoses can be calculated since the DMED provides the number of uniformed personnel serving per year. Incidence data can be grouped by a limited number of demographic categories including sex, age, race, and military service.

Participants included the entire population of active duty military personnel serving between 1997 and 2015. This included SMs in the Army, Navy, Air Force, and Marine Corps. ICD-9 codes used to determine each vitamin deficiency/disorder are shown in Table 1. The number of first occurrences and primary diagnosis in each individual ICD-9 code were obtained by each year, 1997 through 2015, and added together for each category shown in Table 1. Cases involving outpatient visits and hospitalizations (which rarely occurred for these indications) were included and compiled separately. The incidence rate was calculated as the number of SMs with the first incidence of a particular category (grouping) of ICD-9 codes (or individual code in some cases) divided by the population for that year and multiplied by 100,000 (cases/100,000 person-yr [p-yr]). Incidence rate data for the entire cohort were plotted by year.

**Table 1:** ICD-9 Codes for Vitamin Deficiencies & Disorders

Deficiency/Disorder	Specific Nutritional Problems Included in Category (ICD-9 code description)	ICD-9 Code
Thiamine (B_1_) Deficiency	Beriberi	265.0
	Other and Unspecified Thiamine Deficiency	265.1
Riboflavin (B_2_) Deficiency	Ariboflavinosis	266.0
Niacin (B_3_) Deficiency	Pellagra	265.2
Pyridoxine (B_6_) Deficiency	Vitamin B_6_ Deficiency	266.1
Folate (B_9_) Deficiency	Folate Deficiency Anemia	281.2
Vitamin B_12_ Deficiency	Pernicious Anemia (Vitamin B_12_ Malabsorption)	281.0
	“Other Vitamin B_12_” Deficiency Anemia	281.1
Folate & Vitamin B_12_ Deficiency	Other Megaloblastic Anemias	281.3
Other B-Complex Deficiencies	Other B-Complex Deficiencies	266.2
	Unspecified B-Complex Deficiencies	266.9
Vitamin A Deficiency	Vitamin A Deficiency with Conjunctival Xerosis	264.0
	Vitamin A Deficiency with Conjunctival Xerosis & Bitot’s Spot	264.1
	Vitamin A Deficiency with Corneal Xerosis	264.2
	Vitamin A Deficiency with Corneal Ulceration and Xerosis	264.3
	Vitamin A Deficiency with Keratomalacia	264.4
	Vitamin A Deficiency with Night Blindness	264.5
	Vitamin A Deficiency with Xerophthalmic Scars of Cornea	264.6
	Vitamin A Deficiency with Other Ocular Manifestations	264.7
	Vitamin A Deficiency, Other Manifestations	264.8
	Vitamin A Deficiency Unspecified	264.9
	Conjunctival Xerosis Due to Vitamin A Deficiency	372.53
Vitamin C Deficiency	Ascorbic Acid Deficiency	267
Vitamin D Deficiency	Rickets, Active	268.0
	Rickets, Late Effects	268.1
	Osteomalacia, Unspecified	268.2
	Unspecified Vitamin D Deficiency	268.9
Vitamin K Deficiency	Vitamin K Deficiency	269.0
Deficiency of “Other Vitamin”	Deficiency of “Other Vitamins”	269.1
	Unspecified Vitamin Deficiency	269.2
Excess Vitamin A (Hypervitaminosis A)	Hypervitaminosis A	278.2
	Hypercarotinemia	278.3
Excess Vitamin D (Hypervitaminosis D)	Hypervitaminosis D	278.4

Cases were also complied by sex, age (< 20, 20–24, 25–29, 30–34, 35–39, ≥40 years), race (white, black, others), and military service (Army, Navy, Air Force, Marine Corps) with the corresponding population. Populations (N) in each demographic strata were obtained by year from the DMED and descriptive statistics for the entire survey period calculated using the Statistical Package for the Social Sciences (SPSS, Version 26). The incidence rate (cases/100,000 p-yr) for each vitamin deficiency/disorder was calculated for each demographic stratum in each category. A referent stratum for each demographic variable (indicated by an incidence rate ratio = 1.00) was selected and comparisons were made to that stratum. The Open Source Epidemiologic Calculator was used to determine incidence rate ratios with their 95% confidence intervals [22]. Only univariable analyses by demographic characteristic and vitamin deficiency/disorder were conducted. Multivariable analyses were not possible since the DMED only provides data grouped by demographics and does not provide individual data.

## Results

Table 2 shows the demographics of the SM population for the entire period from 1997 through 2015. Table 2 represents averages and standards deviations for all years complied. The population was predominately white men aged 20 to 29 years, although other demographics were also represented. For example, there are over 200,000 women and over 250,000 black SMs.

**Table 2:** Demographics of Service Member Population, 1997–2015

Demographic Variable	Strata	*N* (mean ± SD)	Proportion of Demographic Variable (%)
Total Population		1,382,266 ± 30,987	100.0
Sex	Men	1,181,011 ± 29,728	85.4
	Women	201,255 ± 5378	14.6
Age Group	< 20 yr	102,003 ± 16,856	7.4
	20–24 yr	447,211 ± 23,715	32.4
	25–29 yr	305,918 ± 27,547	22.1
	30–34 yr	210,145 ± 13,502	15.2
	35–40 yr	174,833 ± 18,642	12.6
	> 40 yr	142,156 ± 7758	10.3
Race	White	945,680 ± 28,412	68.4
	Black	250,526 ± 19,488	18.1
	Other	186,060 ± 8247	13.5
Military Service	Army	506,209 ± 31,422	36.6
	Navy	348,099 ± 25,871	25.2
	Air Force	344,083 ± 19,850	24.9
	Marine Corps	183,875 ± 11,663	13.3

Table 3 presents the overall number of cases and incidence rates of each vitamin deficiency and disorder for the entire period. The overall incidence rate of vitamin deficiencies (13 categories) was 92.7 cases/100,000 p-yr and the overall incidence rate for the two hypervitaminosis categories was 0.4 cases/100,000 p-yr. The highest overall rate for a single deficiency was for vitamin D, followed by (in order of decreasing rates) other B-complex deficiency, vitamin B_12_ anemia, deficiencies of “other vitamins”, and vitamin A deficiency. All other deficiencies and disorders had < 1 case/100,000 p-yr. There were very few cases involving hospitalizations (inpatient cases), accounting for only 0.2% (*n* = 57) of the vitamin deficiencies/disorder cases.

**Table 3:** Overall Cases and Incidence Rates for Nutritional Deficiencies and Disorders in US Military Personnel, 1997–2015

Nutritional Deficiency/Disorder	Outpatient		Inpatient		Total	
	Cases (*n*)	Incidence Rate (cases/100,000 person-yr)	Cases (*n*)	Incidence Rate (cases/100,000 person-yr)	Cases (*n*)	Incidence Rate (cases/100,000 person-yr)
Thiamine (B_1_) Deficiency (Beriberi & Other)	209	0.80	12	0.05	221	0.84
Riboflavin (B_2_) Deficiency (Ariboflavinosis)	32	0.12	0	0.00	32	0.12
Niacin (B_3_) Deficiency (Pellagra)	51	0.19	0	0.00	51	0.19
Pyridoxine (B_6_) Deficiency	61	0.23	0	0.00	61	0.23
Folate (B_9_) Anemia	178	0.68	1	0.00	179	0.68
Vitamin B_12_ Anemia	1984	7.55	16	0.06	2000	7.62
Folate and Vitamin B_12_ Anemia	60	0.23	2	0.01	62	0.24
Other B-Complex Deficiencies	5303	20.19	13	0.05	5316	20.24
Vitamin A Deficiency	659	2.51	0	0.00	659	2.51
Vitamin C Deficiency	52	0.20	1	0.00	53	0.20
Vitamin D Deficiency	14,102	53.70	11	0.04	14,113	53.73
Vitamin K Deficiency	49	0.19	0	0.00	49	0.19
Deficiency of “Other Vitamins”	1559	5.94	1	0.00	1560	5.94
Hypervitaminosis A	68	0.26	0	0.00	68	0.26
Hypervitaminosis D	33	0.13	0	0.00	33	0.13

**Fig. 1:**
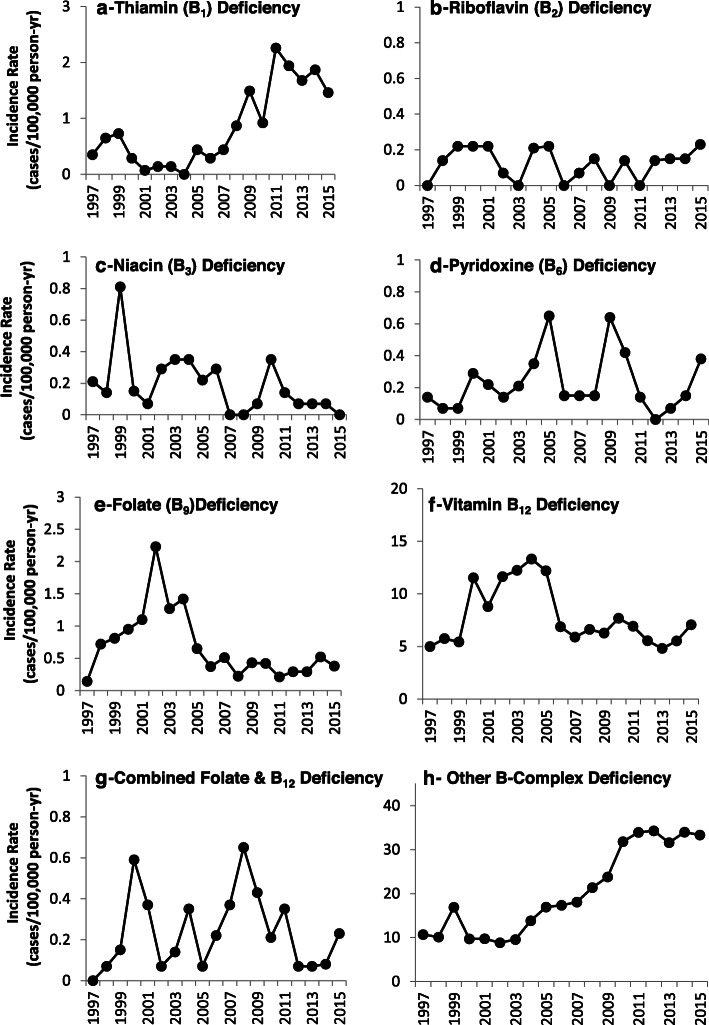
Incidence Rate of Clinically-Diagnosed Vitamin Deficiencies and Disorder in US Military Service Members, 1997–2015

**Fig. 2:**
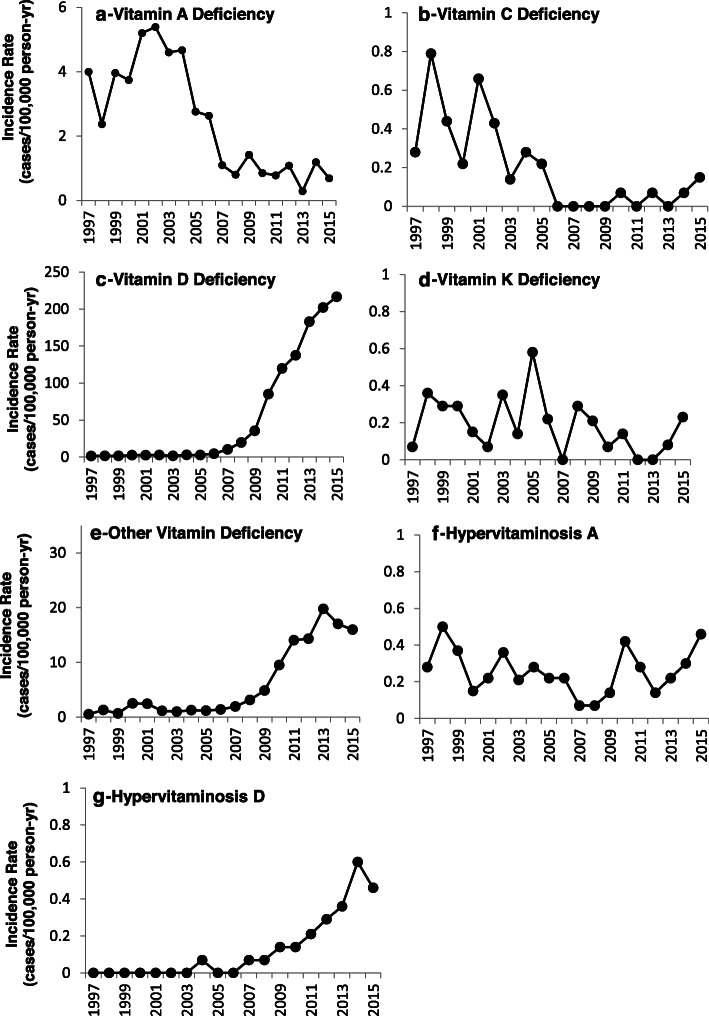
Incidence Rate of Clinically-Diagnosed Vitamin Deficiencies and Disorder in US Military Service Members, 1997–2015

Figure 1 presents secular trends in incidence rates for B-vitamin deficiencies. The vertical axes differ for each vitamin so the temporal trends can be easily visualized. There was little change over years for riboflavin, niacin, pyridoxine, and combined folate and B_12_ deficiencies. Individually, incidence rates for folate and B_12_ deficiencies rose in early years but returned to lower levels later. Thiamine and other B-complex deficiencies were higher in later years. Fig. 2 presents the secular trends in incidence rates for other vitamin deficiencies and the hypervitaminoses. There was little change in rates for vitamin K deficiency and hypervitaminosis A. Vitamin A and vitamin C deficiency incidence rates declined over time. Vitamin D deficiency, “other vitamin” deficiency, and hypervitaminosis D incidence rates rose in later years. Vitamin D deficiency rates ranged from 1.5 to 4.6 cases/100,000 p-yr between 1997 and 2006.

Table 4 presents vitamin deficiencies and disorders by demographic characteristics. Incidence rates for thiamin deficiency were higher among women, blacks, and Army personnel (compared to the Navy and Marines), and generally increased with age. Riboflavin deficiency rates were higher among women, blacks, Army personnel (compared to the Navy), and the oldest age group. Niacin deficiency increased with age and was highest in the Air Force. Pyridoxine deficiency rates were higher among women, older age groups, blacks, and Army personnel (compared to the Navy). Folate deficiency incidence rates were higher among women, the youngest age group (compared to the 20–34 year olds), blacks, and the Army (compared to the Marines). Vitamin B_12_ anemia incidence rates were higher among women, increased with age, were higher among blacks, and were lower in the Navy and Marines, but higher in the Air Force compared to Army. Combined folate and B_12_ deficiency anemia rate was higher among women, but did not differ significantly by age, race, or service. Other B-complex deficiency incidence rates were higher among women, increased substantially with age, and were higher among blacks, but lower in other races (compared to whites), and higher in Army personnel compared to Navy and Marine personnel.

**Table 4:** Univariable Analysis of Vitamin Deficiencies and Disorders among United States Military Personnel by Demographic Characteristics (1997–2015)

Deficiency/Disorder	Variable	Strata	*N*	Incidence Rate (cases/100,000 person-yr)	Incidence Rate Ratio (95% Confidence Interval)	Deficiency/Disorder	Variable	Strata	*N*	Incidence Rate (cases/ 100,000 person-yr)	Incidence Rate Ratio (95% Confidence Interval)
Thiamine (B_1_) Deficiency	Sex	Male	161	0.27	1.00	Riboflavin (B_2_) Deficiency	Sex	Male	19	0.08	1.00
		Female	60	1.57	2.19 (1.63–2.94)			Female	13	0.34	4.05 (1.98–8.13)
	Age	< 20	6	0.31	1.00		Age	< 20	3	0.15	1.00
		20–24	43	0.51	1.63 (0.70–3.84)			20–24	6	0.07	0.46 (0.11–1.82)
		25–29	34	0.58	1.88 (0.79–4.50)			25–29	2	0.03	0.22 (0.04–1.33)
		30–34	48	1.20	3.88 (1.66–9.07)			30–34	6	0.15	0.97 (0.24–3.88)
		35–39	36	1.08	3.50 (1.48–8.31)			35–39	5	0.15	0.97 (0.23–4.07)
		≥40	54	2.00	6.46 (2.78–15.01)			≥40	10	0.37	2.39 (0.66–8.69)
	Race	White	124	0.69	1.00		Race	White	16	0.09	1.00
		Black	75	1.58	2.28 (1.71–3.04)			Black	11	0.23	2.60 (1.20–5.59)
		Other	22	0.62	0.90 (0.57–1.42)			Other	5	0.14	1.59 (0.58–4.34)
	Service	Army	102	1.06	1.00		Service	Army	20	0.21	1.00
		Navy	42	0.64	0.60 (0.42–0.86)			Navy	2	0.03	0.14 (0.03–0.62)
		Air Force	62	0.95	0.89 (0.65–1.23)						
								Air Force	7	0.11	0.51 (0.22–1.22)
								Marines	3	0.09	0.41 (0.13–1.39)
		Marines	15	0.43	0.40 (0.24–0.70)						
Niacin (B_3_) Deficiency	Sex	Male	46	0.21	1.00	Pyridoxine (B_6_) Deficiency	Sex	Male	40	0.18	1.00
		Female	5	0.13	0.64 (0.25–1.61)			Female	21	0.55	3.08 (1.82–5.22)
	Age	< 20	1	0.05	1.00		Age	< 20	0	0.00	–
		20–24	8	0.09	1.83 (0.23–14.59)			20–24	12	0.14	1.00
		25–29	11	0.19	3.67 (0.47–28.41)			25–29	8	0.14	0.97 (0.40–2.38)
		30–34	9	0.23	4.37 (0.55–34.48)			30–34	9	0.23	1.60 (0.67–3.79)
		35–39	8	0.24	4.67 (0.58–37.31)			35–39	13	0.39	2.77 (1.27–6.07)
		≥40	14	0.52	10.05 (1.32–76.38)			≥40	19	0.70	4.98 (2.42–10.26)
	Race	White	36	0.20	1.00		Race	White	33	0.18	1.00
		Black	9	0.19	0.94 (0.45–1.96)			Black	21	0.44	2.40 (1.39–4.15)
		Other	6	0.17	0.85 (0.36–2.01)			Other	7	0.20	1.08 (0.48–2.44)
	Service	Army	14	0.15	1.00		Service	Army	31	0.32	1.00
		Navy	10	0.15	1.03 (0.46–2.34)			Navy	8	0.12	0.38 (0.17–0.82)
		Air Force	22	0.34	2.31 (1.18–4.52)			Air Force	16	0.24	0.76 (0.42–1.39)
		Marines	5	0.14	0.98 (0.35–2.73)			Marines	6	0.17	0.53 (0.22–1.28)
Folate (B_9_) Deficiency Anemia	Sex	Male	80	0.36	1.00	Vitamin B_12_ Anemia	Sex	Male	1147	5.11	1.00
		Female	99	2.59	7.26 (5.41–9.75)			Female	853	22.31	4.36 (3.99–4.77)
	Age	< 20	20	1.03	1.00		Age	< 20	59	3.04	1.00
		20–24	46	0.54	0.52 (0.31–0.89)			20–24	275	3.24	1.06 (0.80–1.41)
		25–29	28	0.48	0.47 (0.26–0.83)			25–29	276	4.75	1.56 (1.18–2.07)
		30–34	18	0.45	0.43 (0.23–0.83)			30–34	297	7.44	2.44 (1.85–3.23)
		35–39	29	0.87	0.85 (0.48–1.50)			35–39	420	12.64	4.15 (3.16–5.45)
		≥40	38	1.41	1.36 (0.79–2.34)			≥40	673	24.92	8.19 (6.27–10.68)
	Race	White	92	0.51	1.00		Race	White	1144	6.37	1.00
								Black	647	13.59	2.14 (1.94–2.35)
		Black	74	1.55	3.04 (2.24–4.12)			Other	209	5.91	0.93 (0.80–1.08)
		Other	13	0.37	0.72 (0.40–1.28)						
	Service	Army	81	0.84	1.00		Service	Army	820	8.53	1.00
		Navy	42	0.64	0.75 (0.52–1.10)			Navy	424	6.41	0.75 (0.67–0.85)
								Air Force	634	9.70	1.14 (1.03–1.26)
		Air Force	43	0.66	0.78 (0.54–1.13)			Marines	122	3.49	0.41 (0.34–0.50)
		Marines	13	0.37	0.44 (0.25–0.79)						
Folate & Vitamin B_12_ Anemia	Sex	Male	43	0.19	1.00	Other B-Complex Deficiency	Sex	Male	3062	13.65	1.00
		Female	19	0.50	2.59 (1.51–4.45)			Female	2254	58.95	4.32 (4.09–4.56)
	Age	< 20	5	0.26	1.00		Age	< 20	87	4.49	1.00
		20–24	9	0.11	0.41 (0.14–1.23)			20–24	678	7.98	1.78 (1.42–2.22)
		25–29	13	0.22	0.87 (0.31–2.43)			25–29	799	13.75	3.06 (2.45–3.82)
		30–34	6	0.15	0.58 (0.18–1.91)			30–34	861	21.57	4.80 (3.85–5.99)
		35–39	12	0.36	1.40 (0.49–3.97)			35–39	1096	33.00	7.35 (5.91–9.14)
		≥40	17	0.63	2.44 (0.90–6.61)			≥40	1795	66.46	14.80 (11.94–18.36)
	Race	White	39	0.22	1.00		Race	White	3371	18.76	1.00
		Black	17	0.36	1.65 (0.93–2.91)			Black	1348	28.32	1.51 (1.42–1.61)
		Other	6	0.17	0.78 (0.33–1.85)			Other	597	16.89	0.90 (0.83–0.98)
	Service	Army	22	0.23	1.00		Service	Army	2364	24.58	1.00
		Navy	14	0.21	0.92 (0.47–1.81)			Navy	1023	15.47	0.63 (0.58–0.68)
		Air Force	21	0.32	1.40 (0.77–2.55)			Air Force	1642	25.12	1.02 (0.96–1.09)
		Marines	5	0.14	0.63 (0.24–1.65)			Marines	287	8.21	0.33 (0.30–0.38)
Vitamin A Deficiency	Sex	Male	4367	1.95	1.00	Vitamin C Deficiency	Sex	Male	41	0.18	1.00
		Female	222	5.81	2.98 (2.53–3.50)			Female	12	0.31	1.72 (0.90–3.27)
	Age	< 20	82	4.23	1.00		Age	< 20	5	0.26	1.00
		20–24	205	2.41	0.57 (0.44–0.74)			20–24	20	0.24	0.91 (0.34–2.43)
		25–29	116	2.00	0.47 (0.36–0.63)			25–29	7	0.12	0.47 (0.15–1.47)
		30–34	83	2.08	0.49 (0.36–0.67)			30–34	5	0.13	0.49 (0.14–1.68)
		35–39	83	2.50	0.59 (0.44–0.80)			35–39	7	0.21	0.82 (0.26–2.57)
		≥40	90	3.33	0.78 (0.58–1.06)			≥40	9	0.33	1.29 (0.43–3.85)
	Race	White	382	2.13	1.00		Race	White	41	0.23	1.00
		Black	176	3.70	1.74 (1.46–2.08)			Black	9	0.19	0.83 (0.40–1.71)
		Other	101	2.86	1.34 (1.08–1.67)			Other	3	0.08	0.37 (0.12–1.20)
	Service	Army	211	2.19	1.00		Service	Army	15	0.16	1.00
		Navy	126	1.91	0.87 (0.70–1.08)			Navy	19	0.29	1.84 (0.95–3.63)
		Air Force	266	4.07	1.86 (1.55–2.22)			Air Force	14	0.21	1.37 (0.66–2.84)
		Marines	56	1.60	0.73 (0.54–0.98)			Marines	5	0.14	1.29 (0.47–3.54)
Vitamin D Deficiency	Sex	Male	9039	40.28	1.00	Vitamin K Deficiency	Sex	Male	35	0.16	1.00
		Female	5074	132.70	3.29 (3.18–3.41)			Female	14	0.37	2.35 (1.26–4.36)
	Age	< 20	514	26.52	1.00		Age	< 20	2	0.10	1.00
		20–24	1957	23.03	0.87 (0.79–0.96)			20–24	16	0.19	1.83 (0.42–7.94)
								25–29	9	0.15	1.50 (0.32–6.94)
		25–29	2542	43.74	1.65 (1.50–1.81)			30–34	4	0.10	0.97 (0.18–5.30)
		30–34	2380	59.61	2.25 (2.04–2.47)			35–39	6	0.18	1.75 (0.35–8.67)
		35–39	2636	79.36	2.99 (2.72–3.29)			≥40	12	0.44	4.31 (0.96–19.23)
		≥40	4084	151.22	5.70 (5.20–6.25)						
	Race	White	7946	44.22	1.00		Race	White	34	0.19	1.00
								Black	10	0.21	1.11 (0.55–2.25)
		Black	4099	86.11	1.95 (1.88–2.02)			Other	5	0.14	0.75 (0.29–1.91)
		Other	2068	58.50	1.32 (1.26–1.39)						
	Service	Army	6566	68.27	1.00		Service	Army	22	0.23	1.00
		Navy	2968	44.87	0.66 (0.63–0.69)			Navy	8	0.12	0.53 (0.24–1.19)
		Air Force	3602	55.10	0.81 (0.77–0.84)			Air Force	16	0.24	1.07 (0.56–2.04)
		Marines	977	27.96	0.41 (0.38–0.44)			Marines	3	0.09	0.38 (0.11–1.25)
Deficiencies of “Other Vitamins”	Sex	Male	951	4.24	1.00	Hyper-vitaminosis A	Sex	Male	46	0.21	1.00
		Female	609	15.93	3.76 (3.39–4.16)			Female	22	0.58	2.81 (1.69–4.66)
	Age	< 20	38	1.96	1.00		Age	< 20	2	0.10	1.00
		20–24	219	2.58	1.31 (0.93–1.86)			20–24	18	0.21	2.05 (0.48–8.85)
		25–29	292	5.02	2.56 (1.83–3.59)						
								25–29	10	0.17	1.67 (0.37–7.61)
		30–34	240	6.01	3.07 (2.18–4.32)			30–34	11	0.28	2.67 (0.59–12.04)
								35–39	13	0.39	3.79 (0.86–16.8)
		35–39	280	8.43	4.30 (3.06–6.03)			≥40	14	0.52	5.02 (1.14–22.10)
		≥40	491	18.18	9.27 (6.67–12.90)						
	Race	White	902	5.02	1.00		Race	White	47	0.26	1.00
		Black	465	9.77	1.95 (1.74–2.18)			Black	15	0.32	1.21 (0.67–2.15)
		Other	193	5.46	1.09 (0.93–1.27)			Other	6	0.17	0.65 (0.28–1.52)
	Service	Army	762	7.92	1.00		Service	Army	20	0.21	1.00
		Navy	290	4.38	0.55 (0.48–0.63)			Navy	22	0.33	1.60 (0.87–2.93)
		Air Force	413	6.32	0.80 (0.71–0.90)			Air Force	22	0.34	1.62 (0.88–2.97)
		Marines	95	2.72	0.34 (0.28–0.42)			Marines	4	0.11	0.55 (0.19–1.61)
Hyper-vitaminosis D	Sex	Male	19	0.08	1.00						
		Female	14	0.37	4.32 (2.17–8.62)						
	Age	< 20	1	0.05	1.00						
		20–24	5	0.06	1.14 (0.13–9.76)						
		25–29	7	0.12	2.33 (0.29–18.97)						
		30–34	8	0.20	3.88 (0.49–31.04)						
		35–39	5	0.15	2.92 (0.34–24.97)						
		≥40	7	0.26	5.02 (0.62–40.82)						
	Race	White	24	0.13	1.00						
		Black	9	0.19	1.42 (0.66–3.05)						
		Other	0	0.00	–						
	Service	Army	16	0.17	1.00						
		Navy	5	0.08	0.45 (0.17–1.24)						
		Air Force	11	0.17	1.01 (0.47–2.18)						
		Marines	1	0.03	0.17 (0.02–1.30)						

Vitamin A deficiency incidence rates were higher among women, the youngest age group (compared to those 20–39 years of age), blacks and other races, and in Air Force personnel, but lower in Marines (compared to the Army). Vitamin C deficiency incidence rates differed little by demographic characteristics. Vitamin D deficiency incidence rates were higher in women, generally increased with age, were higher among blacks and other races, and higher among Army personnel compared to other services. Vitamin K deficiency incidence rates were higher in women, but differed little by other demographics. The incidence rates for deficiencies of “other vitamins” were higher in women, increased with age, were higher for blacks, and were higher in Army personnel compared to the other services. Hypervitaminosis A incidence rates were higher among women and tended to increase with age. Hypervitaminosis D incidence rates were higher among women, but differed little by other demographics.

## Discussion

This study was, to our knowledge, the first examination of incidence rates of clinically-diagnosed vitamin deficiencies and disorders in the entire US military population, or any other US population. The overall rates were low, but the likelihood of a diagnosis of any specific deficiency varied substantially. Rates also varied by demographic characteristics, with women and blacks typically having higher incidences of many deficiencies. These findings can guide clinical decision-making with regard to testing for specific deficiencies in at risk military and similar civilian populations and delivering public health information to specific demographic groups. Although the demographics of the military population varies from the overall US population, it is likely these findings apply to the younger portions of the US civilian population and have similar clinical and public health implications.

One of the most dramatic trends observed was a large increase in the incidence rate for vitamin D deficiencies beginning about 2007. Prescriptions written by health care providers for vitamin D and filled by SMs increased 55-fold from 2005 to 2013 [20], and prescription fill rates were higher among women, increased with age, and were highest in the Army and lowest in the Marines [23], corresponding with the data presented here. In 1997 (the beginning of our survey period), an Institute of Medicine (IOM) report established the adequate intake level for vitamin D at 200 International Units (IU) for adults based on the amount of dietary intake required to achieve a serum level of plasma 25(OH) D level of about 30 nmols/l [24]. Subsequently, additional research indicated either the plasma 25(OH) D levels or the dietary intake of vitamin D of Americans were insufficient [25, 26]. This was followed by considerable media attention [27–29] and scientific/medical reporting [7, 30, 31]. More accurate methods of measuring 25(OH) D also became available [32]. In 2011, the IOM concluded 25(OH) D levels < 30 nmols/l were associated with reduced calcium absorption and osteomalacia in young and middle aged adults, but there was little evidence of benefits for levels > 50 nmols/l [33]. To achieve a plasma level of 50 nmols/l, the committee recommended a daily allowance (RDA) of vitamin D of 600 IU/day [33]. The increasing attention devoted to vitamin D in the popular and medical literature, better availability and more accurate assay procedures, knowledge that large portions of individuals may be vitamin D deficient, and the change in national policy may account for the increase in vitamin D deficiency diagnoses during the survey period.

Coincident with the rise in vitamin D deficiencies was an increase in vitamin D hypervitaminosis. There was ≤1 case/year prior to 2008 with a maximum of 8 cases in 2014 so overall rates were small. As testing for vitamin D deficiency increased, it is not surprising vitamin D hypervitaminosis increased. One population-based study found 1.3% of individuals tested had 25(OH) D levels > 125 nmol/l [34]. Some of the vitamin D hypervitaminosis cases observed in this study could be associated with use of vitamin D dietary supplements since 69% of SMs use supplements [35] and many contain vitamin D. Vitamin D has been reported to have ergogenic effects, which may encourage SMs to use vitamin D supplements [5, 36]. Dosages on supplement labels are often inconsistent with actual content which can lead to high/excess intakes in some cases [37].

Other interesting secular trends were the decreases in clinically-diagnosed vitamins A and C deficiency over time, although the incidence rates were low overall. Prescriptions filled for vitamin C supplements by SMs increased in the 2005–2013 period [20], possibly accounting for at least a portion of the decline in vitamin C deficiency. In 2000, the RDA of vitamin A was lowered [38, 39] and coincident with this was the decline in vitamin A deficiency diagnoses a few years later among SMs. The lower vitamin A RDA and setting of an upper tolerable limit raised some concern about excessive intake [40, 41], but as shown here the incidence rates for hypervitaminosis A was extremely low and changed little during the survey period in the military population.

The incidence rates for other B-complex vitamins and “other vitamin” deficiencies rose over the survey period. Other B-complex and “other vitamin” deficiencies respectively accounted for 22% (5316/24,356) and 6% (1560/24,356) of the vitamin deficiency cases, respectively. Clinical experience suggests medical care providers use ICD-9 codes associated with these “other” categories when a specific deficiency is not listed within the ICD-9 codes, and also when they cannot determine a specific vitamin that might be associated with a medical problem. If the latter is the case, it may be that the incidence rates for some specific clinically diagnosed vitamin deficiencies may be underestimated, but this cannot be determined with certainty given the data available within the DMED.

Vitamin B_12_ anemia had the third highest incidence rate in this study, increased between 1998 and 2005 and returning to lower levels after 2005. Data from NHANES [42] showed little change in *serum* levels of vitamin B_12_ from 1988 to 2004 and little difference by sex. Serum levels did decrease with age [42] and lower intake levels or intakes below the RDA were more predominant in older adults and non-Hispanic blacks (compared to non-Hispanic whites) [43, 44], which corresponds with the clinical data in the present study.

Various demographic factors were associated with higher deficiency/disorder rates. Women had higher rates than men in 13 of the 15 deficiency/disorder categories examined. Data from NHANES (2003–2008) indicated women were more likely than men to have insufficient intakes of folate, vitamin B_12_, and vitamin D [43, 45]. Women may be more likely to be diagnosed with various illnesses since they use more medical care than men [23, 46–48] even after excluding visits for pregnancy [46, 48]. Age was also associated with vitamin deficiencies/disorders since 9 of 13 vitamin deficiency/disorder categories (thiamin, niacin, pyridoxine, vitamin B_12_, other B-complex, vitamin D, “other vitamins”, and hypervitaminoses A and D) increased with age. Data from NHANES (2009–2012) showed the proportion of individuals below the Estimated Average Requirement increased with age for many B-vitamins (thiamin, niacin, folate, pyridoxine, B_12_) even when dietary supplement intake was included [49], an important factor in military personnel given their high use of dietary supplements compared to civilians [35]. Vitamins C and D did not show the age-related trend [49]. Also, previous studies show that for most major diagnostic categories, ambulatory visits and filling of drug prescriptions are higher among older individuals compared to younger ones [23, 46], evidence of greater health care use with aging. With regard to race, blacks had higher disorder/deficiency rates in 10 of 15 categories. Data from NHANES (2009–2012) indicates a larger proportion of non-Hispanic blacks consumed less than their estimated average requirement for thiamin, riboflavin, niacin, folate, pyridoxine, vitamin B_12_, and vitamin D [44] or had insufficient intakes of many of these vitamins compared to non-Hispanic whites [45]. Race may be associated with behaviors that favor certain types of foods or preparation methods that might influence vitamin intake [50–52].

There are limitations that should be considered in interpreting the findings of this study. Medical care provided by medics and at deployed locations may not be included in the DMED, although it is unlikely the nutritional conditions examined in this study would be diagnosed by medics or in the field. Also, our analysis is based on the primary diagnosis of the nutritional condition (first listed condition) in the DMED and thus may not account for comorbidities. The DMED only provides the first case of SM medical problem by ICD-9 code (incidence) or the total number of visits for that medical condition (total encounters). If the nutritional condition (ICD-9 code) was listed in position other than the first (primary) because of a comorbid problem that condition would not be included in the analysis. Thus, this analysis is limited to the nutritional conditions for which the diagnoses were the primary ones. Finally, the rates here are undoubtedly an underestimate of the actual incidence of these deficiencies/disorders in the military population. The data only include cases where SMs presented to a medical care provider and the provider diagnosed the patient as having a specific deficiency/disorder. It is likely that a number of individuals who had deficiencies did not seek medical attention.

## Conclusions

This study found a low rate of clinically-diagnosed vitamin deficiencies/disorders. Despite this, vitamin D deficiency, deficiency of other B-complex vitamins, and deficiencies of “other vitamins” had increasing incidence rates and are areas of concern that should be under continued surveillance. Women had higher incidence rates for 13 of 15 deficiency/disorder categories and blacks had higher incidence rates in 10 of 15 categories. While we are unaware of any other population-based data assessment of medical records to assess incidence of diagnosed dietary deficiencies and disorders, given advancement in digitized medical records and the desire to access and use large data sets it is likely that such information could be available in the US population in the near future. As such, our results will be useful to those conducting these future analyses.

## Data Availability

Available from authors on reasonable request

## Abbreviations

25(OH)D: 25-hydroxyvitamin D
DMED: Defense Medical Epidemiology Database
ICD-9: International Classification of Diseases, Revision 9
IOM: Institute of Medicine
IU: International units
NHANES: National Health and Nutrition Examination Survey
p-yr: Person-years
RDA: Recommended daily allowance
SM: Service member
US: United States
